# Supplementary material for: Chloroquine-Based Mitochondrial ATP Inhibitors
Source: Molecules. 2023 Jan 24;28(3):1161. doi: 10.3390/molecules28031161 (PMC9920964; doi:10.3390/molecules28031161)
Supplement: Supplementary file 1 [file molecules-28-01161-s001.zip › molecules-2139888-supplementary.pdf]

Chloroquine-based mitochondrial ATP inhibitors

Zhiguo Wang,[†] Robert J. Sheaff,*[, †] and Syed R. Hussaini*[, †]

[†]Department of Chemistry and Biochemistry, The University of Tulsa, 430 South Gary Place, Tulsa, Oklahoma 74104, United States

Email: syed-hussaini@utulsa.edu

Table of Contents

1.	Experimental procedures for the synthesis of **9** and **10**	S2
2.	NMR spectra of isolated compounds	S3–S15
3.	Biochemical data	S16
4.	References	S17

5-Iodopentan-2-one (**9**)[1]

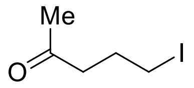

Hydriodic acid (57% in H_2O, 1.25 mL, 9.48 mmol) and α-acetylbutyrolactone (1.00 mL, 9.19 mmol) were added to a round bottom flask equipped with a condenser and containing a stirring bar. The flask was heated at 80 °C for 30 minutes and then allowed to come to rt. Sodium bicarbonate was added to the reaction mixture until pH =7 was reached. The product was extracted with 3:1 hexanes *tert*-butyl methyl ether (5 mL X 4). The combined organic phase was dried over Na_2SO_4. The solvent was removed with rotary evaporation to afford semi-pure **9** as a brown liquid (1.26 g, 65%). R_f = 0.42 (4:1 hexanes/EtOAc). The ^{1}H and ^{13}C spectra matched the literature report.[1]

5-(Ethyl(hydroxyethyl)amino)pentan-2-one (**10**)[1]

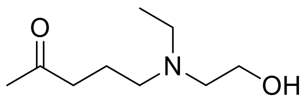

Compound **9** (1.27 g, 5.98 mmol) was dissolved in anhydrous THF (11.0 mL) and added under N_2 to a flask containing K_2CO_3 (4.13 g, 29.6 mmol). 2-(Ethylamino)ethanol (0.658 mL, 6.61 mmol) was added dropwise to the stirred mixture, and the reaction refluxed for 3 hours. The reaction was allowed to come to rt, followed by adding 10.0 mL of saturated aqueous NH_4Cl solution. The reaction was extracted with CH_2Cl_2 (20 mL X 3), and the combined organic layer was dried over Na_2SO_4. The solvent was evaporated, and the crude was purified by vacuum distillation to give pure **10** as a light yellow liquid (831 mg, 4.80 mmol, 80\%). The ^{1}H and ^{13}C spectra matched the reported ones.[1] ^{1}H NMR (400 MHz, $CDCl_3$) δ 3.53 (t, *J* = 5.6 Hz, 2H), 2.90 (s, br, 1H), 2.58 (t, *J* = 5.6 Hz, 2H), 2.55 (q, *J* = 7.2 Hz, 2H), 2.46 (t, *J* = 7.2 Hz, 4H), 2.15 (s, 3H), 1.73 (quin, J = 7.0 Hz, 2H), 1.01 (t, *J* = 7.2 Hz, 3H); ^{13}C NMR (100 MHz, $CDCl_3$) δ 208.7, 58.6, 55.0, 52.4, 47.2, 41.3, 30.0, 21.3, 11.8.

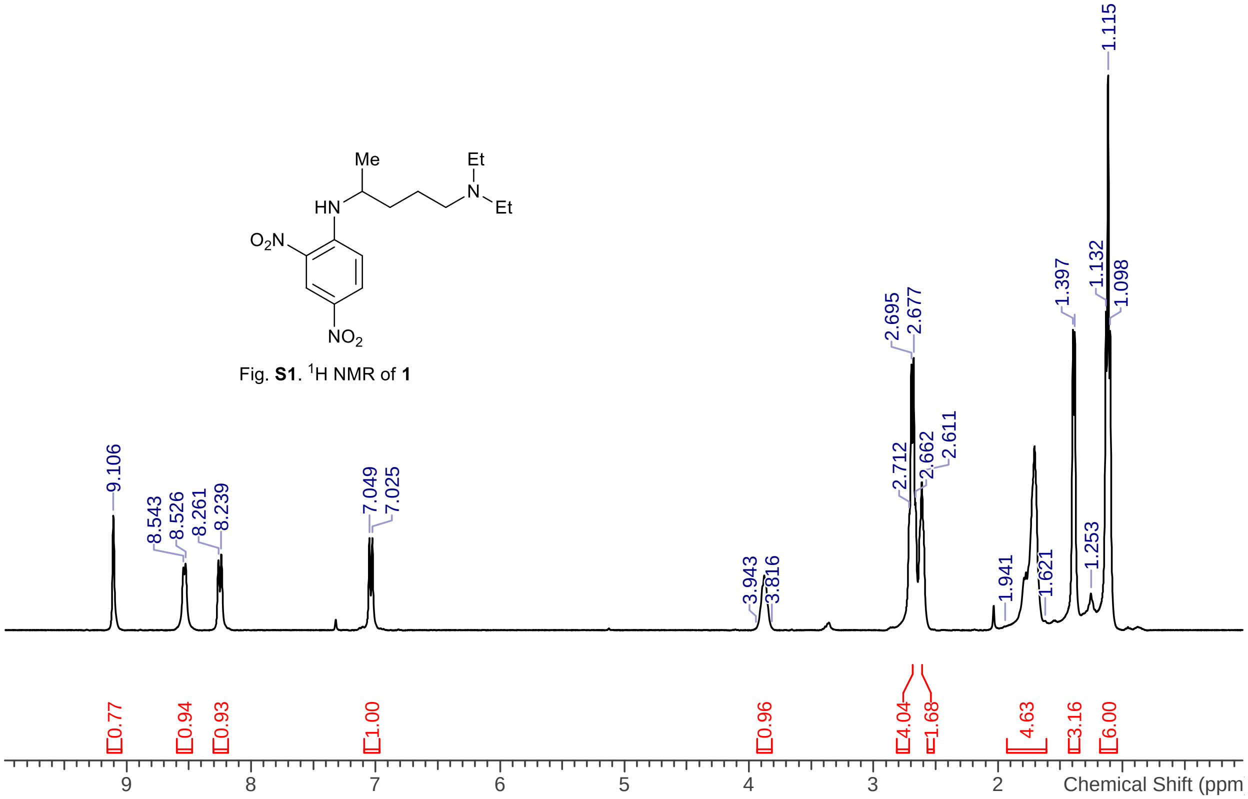

Fig. **S1**. ^{1}H NMR of **1**

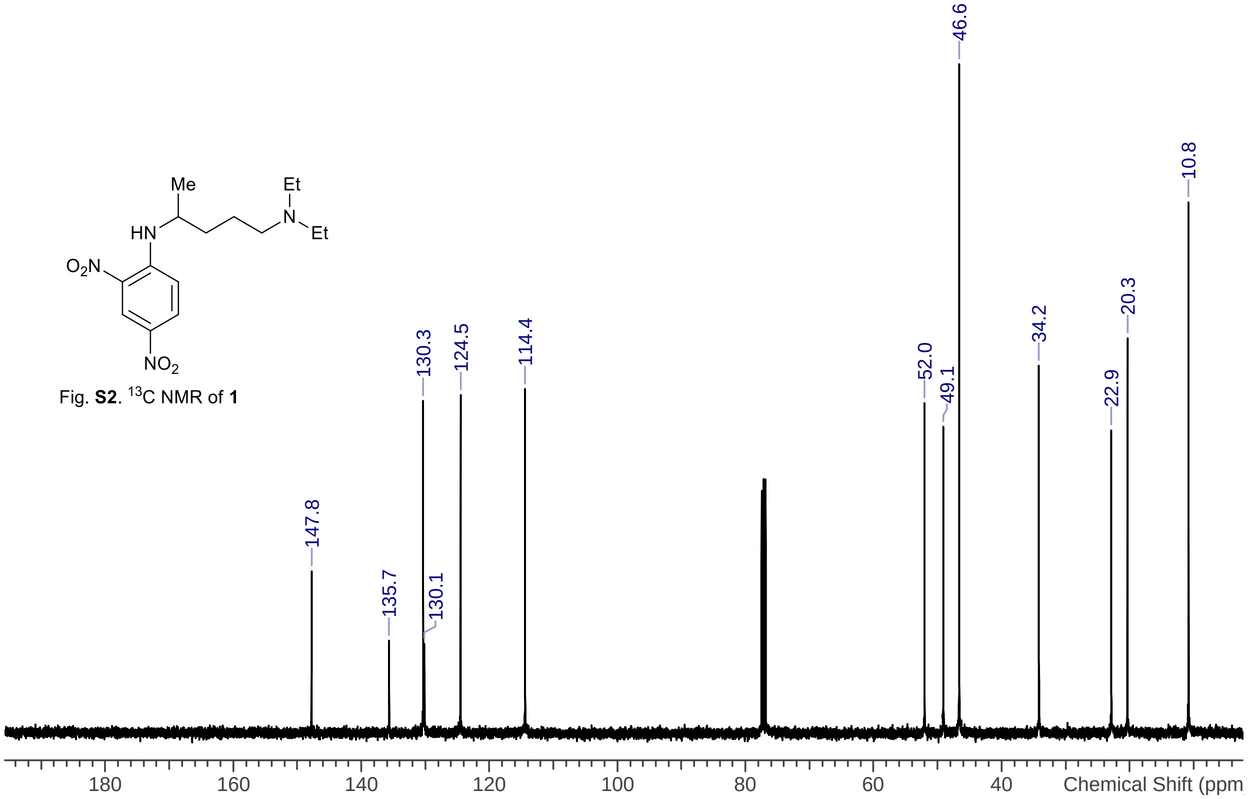

Fig. **S2**. ^{13}C NMR of **1**

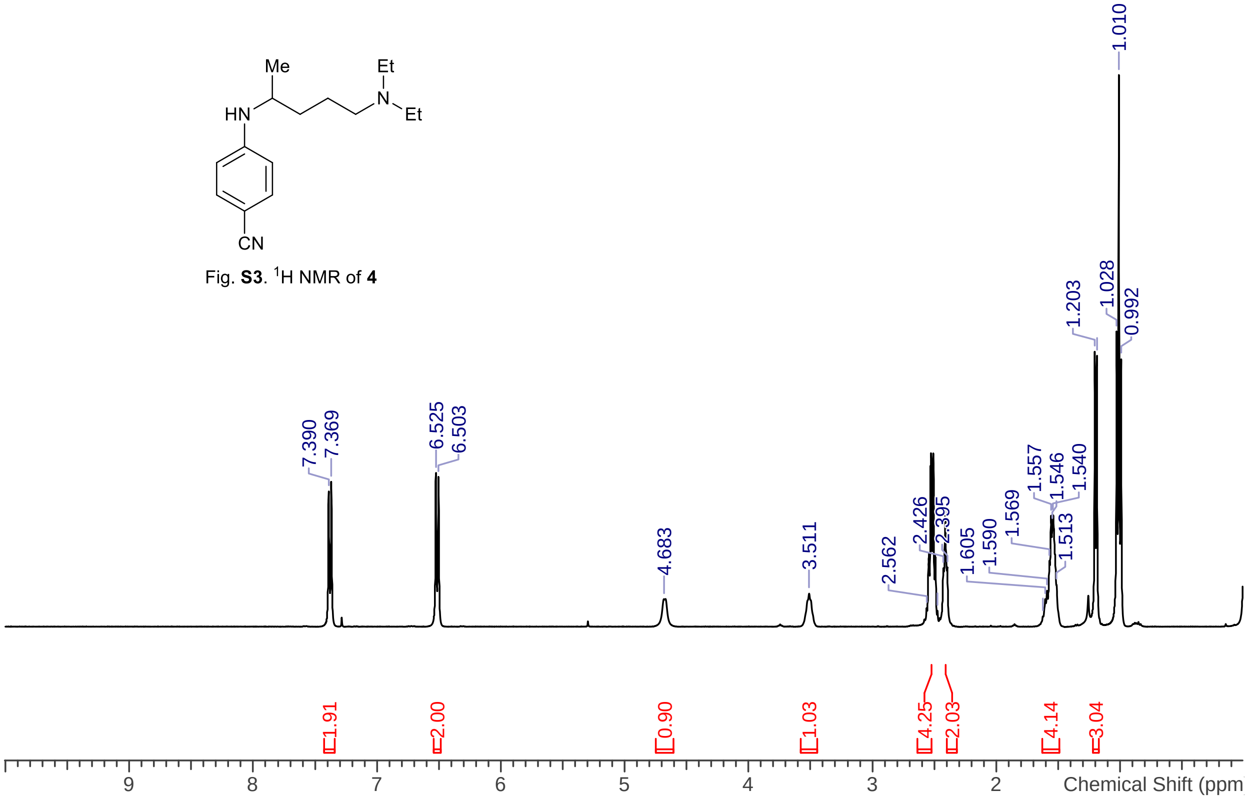

Fig. **S3**. ^{1}H NMR of **4**

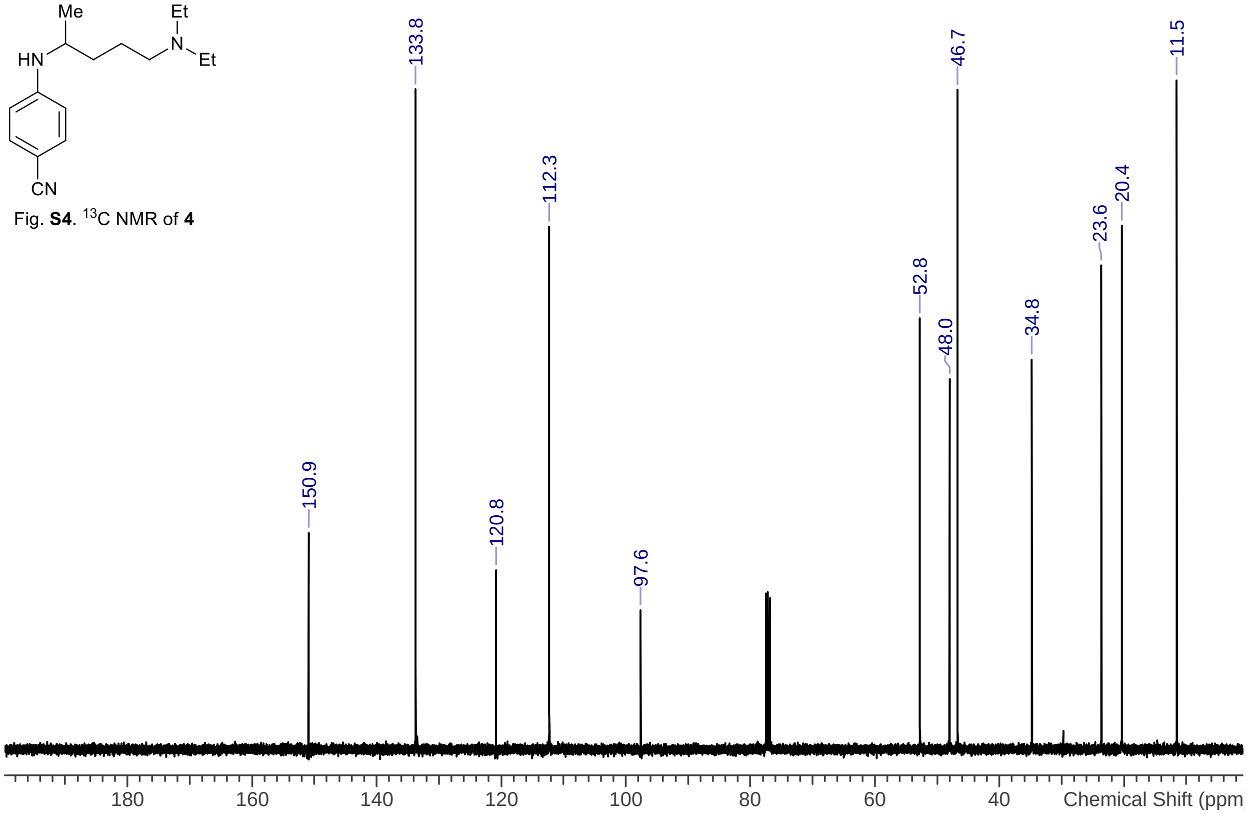

Fig. **S4**. ^{13}C NMR of **4**

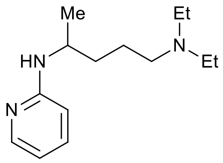

Fig. **S5**. ^{1}H NMR of **5**

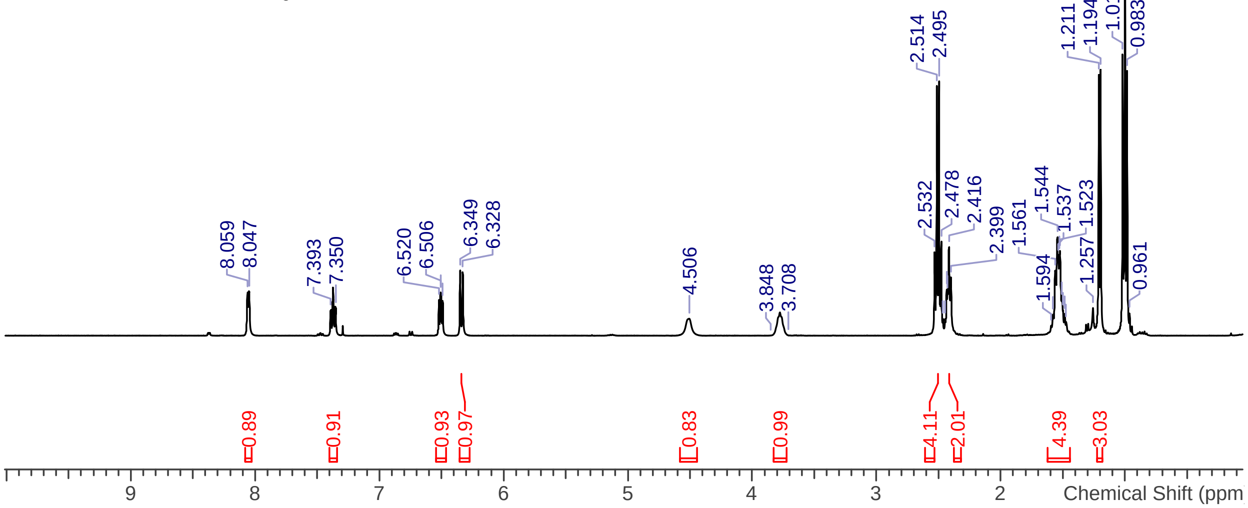

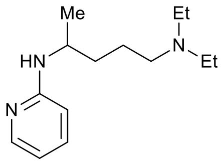

Fig. **S6**. ^{13}C NMR of **5**

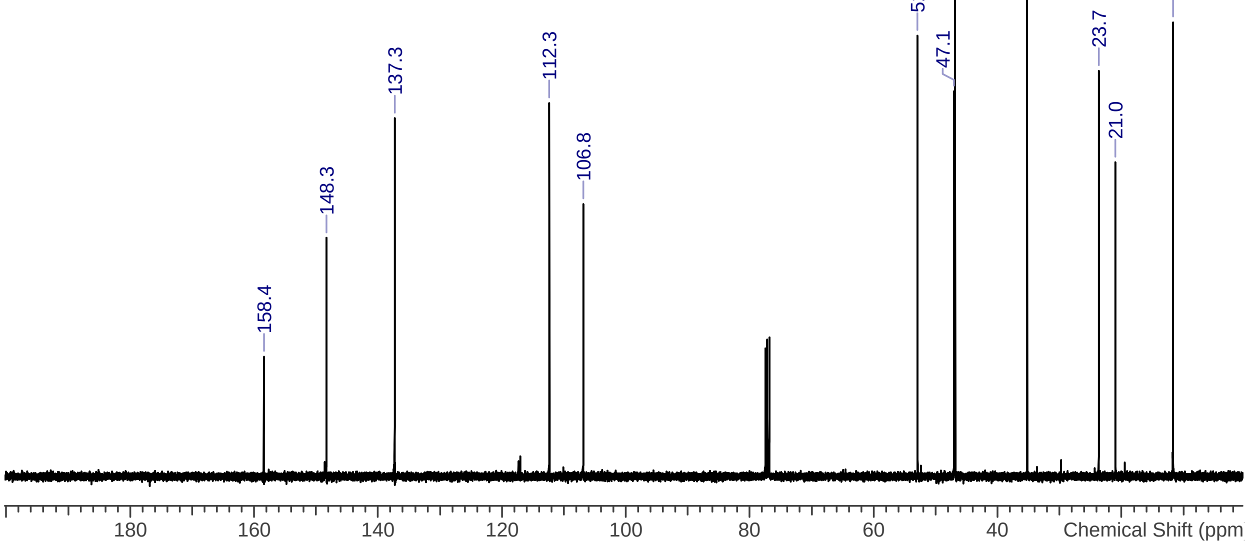

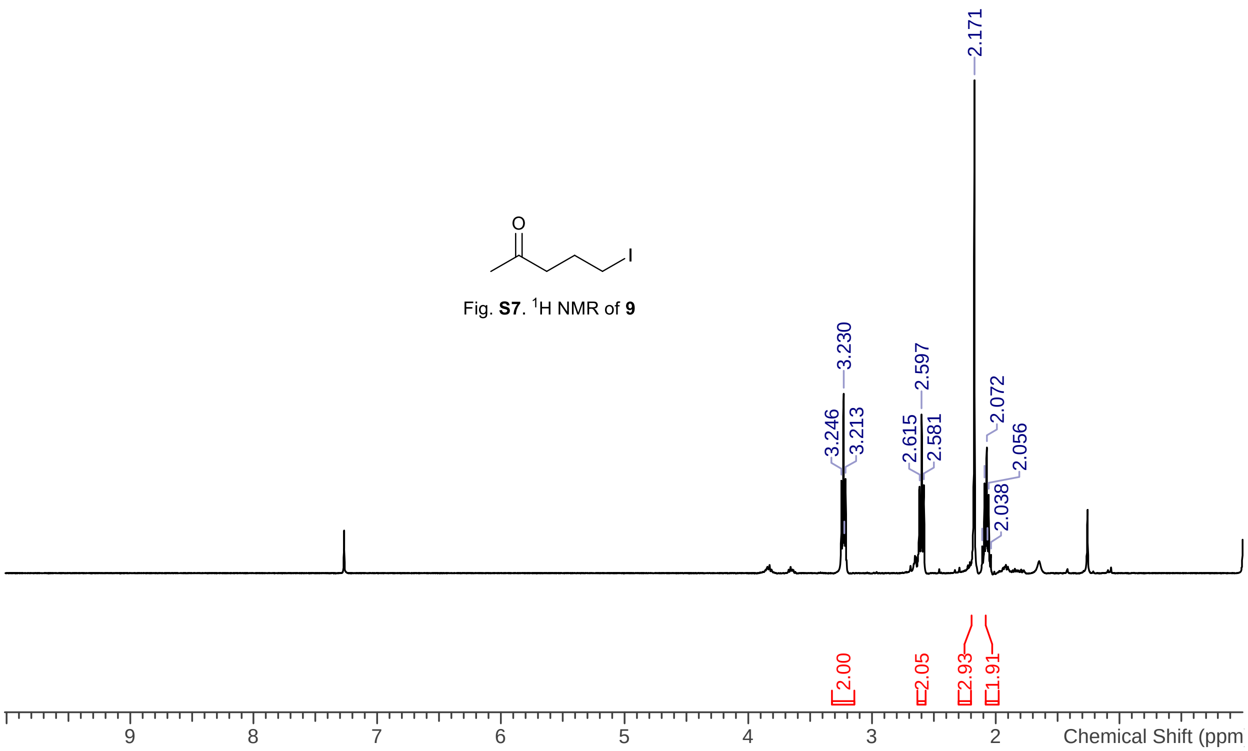

Fig. **S7**. ^{1}H NMR of **9**

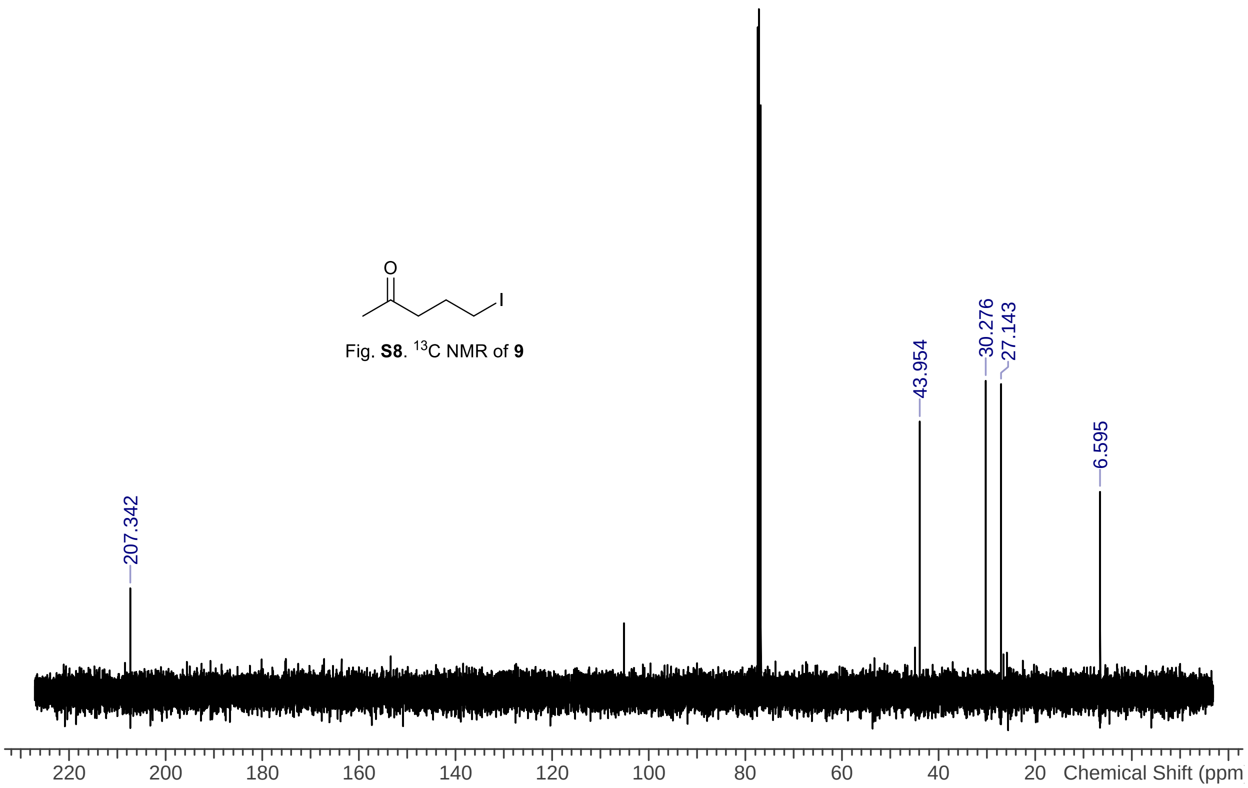

Fig. **S8**. ^{13}C NMR of **9**

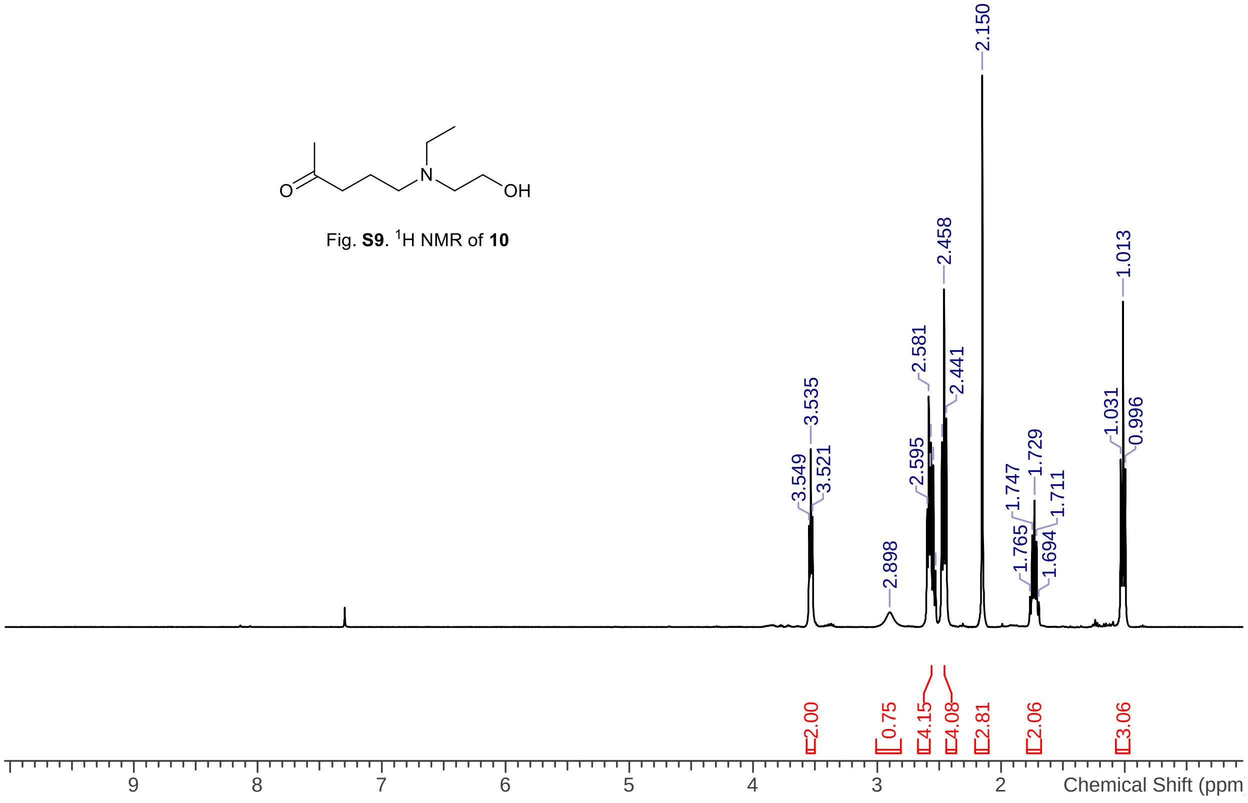

Fig. **S9**. ^{1}H NMR of **10**

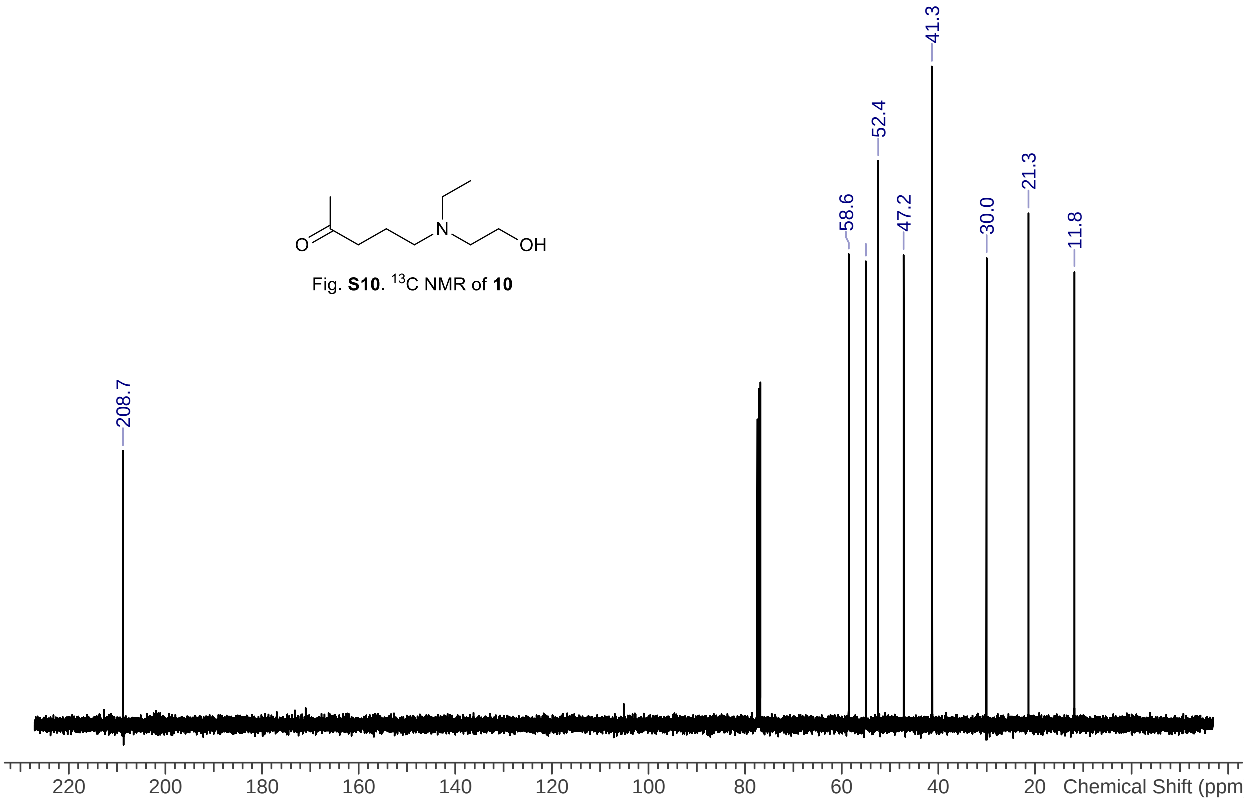

Fig. **S10**. ^{13}C NMR of **10**

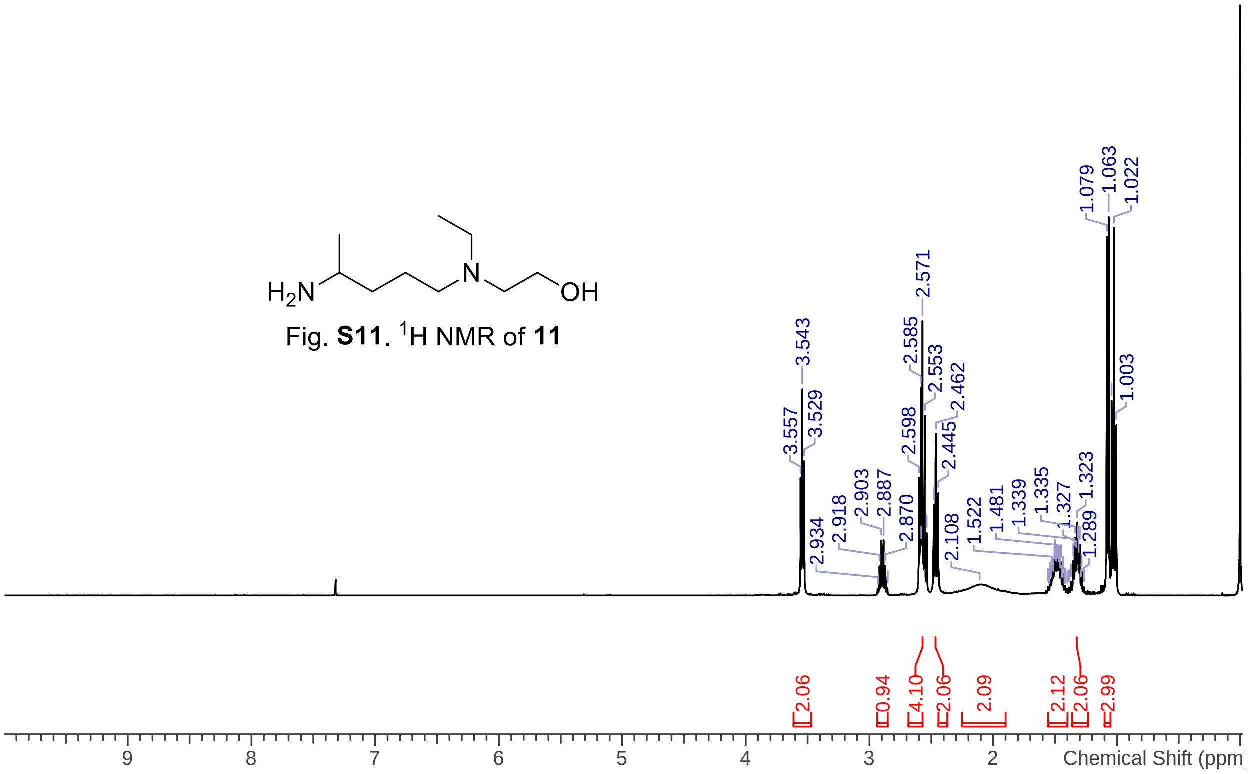

Fig. **S11**. ^{1}H NMR of **11**

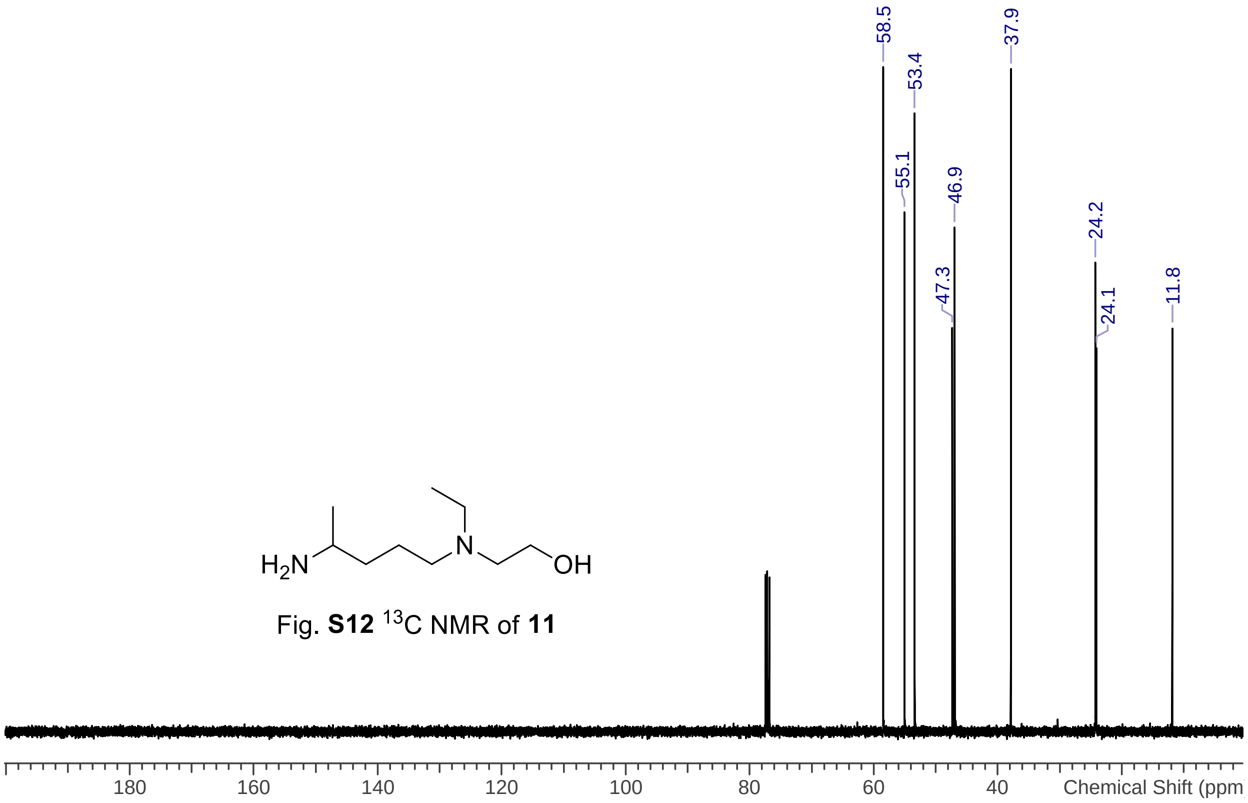

Fig. **S12** ^{13}C NMR of **11**

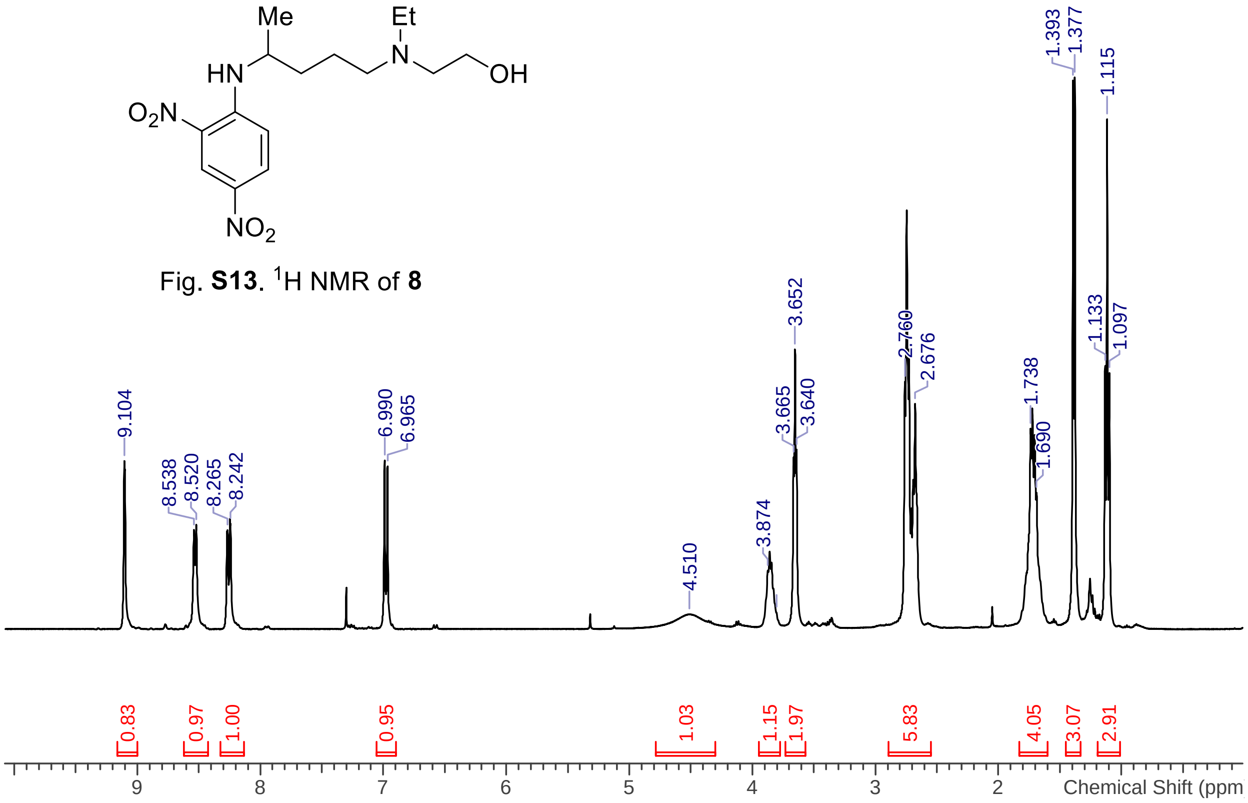

Fig. **S13**. ^{1}H NMR of **8**

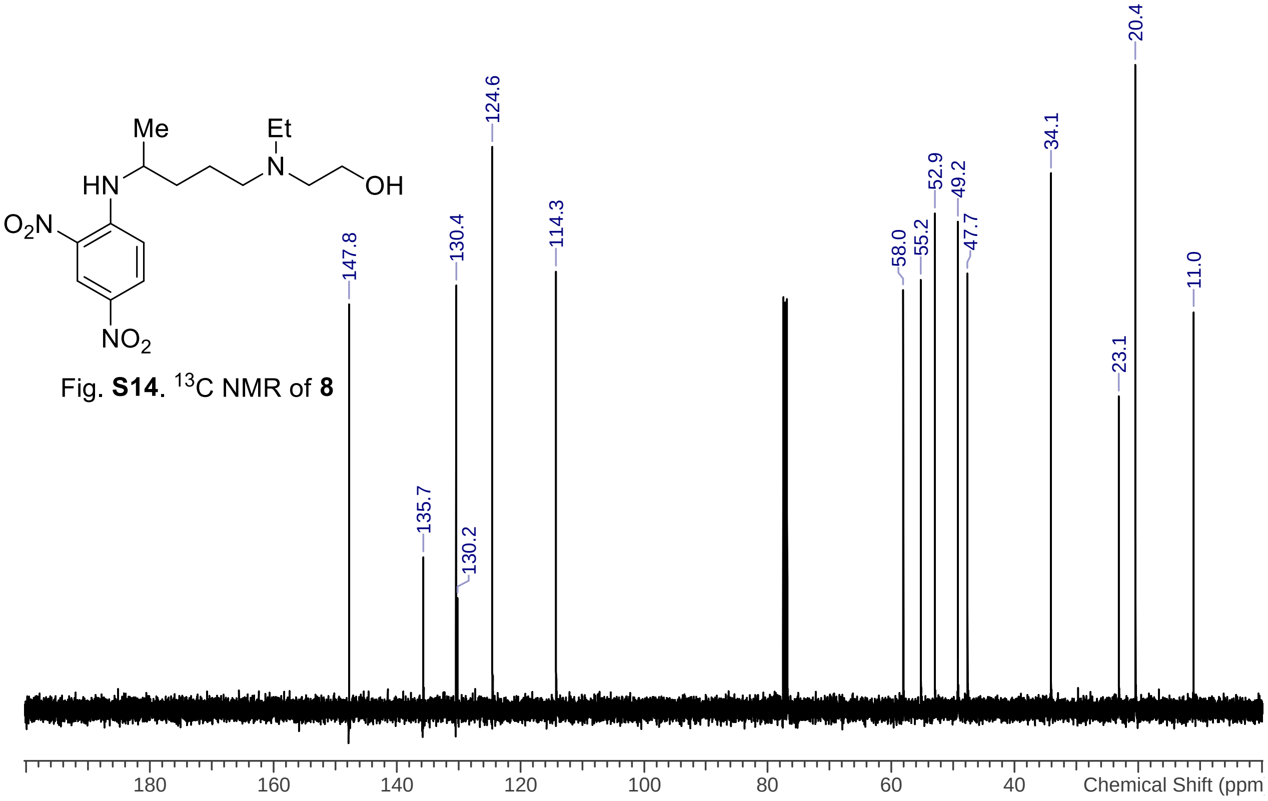

Fig. **S14**. ^{13}C NMR of **8**

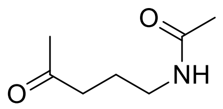

Fig. **S15**. ^{1}H NMR of **12**

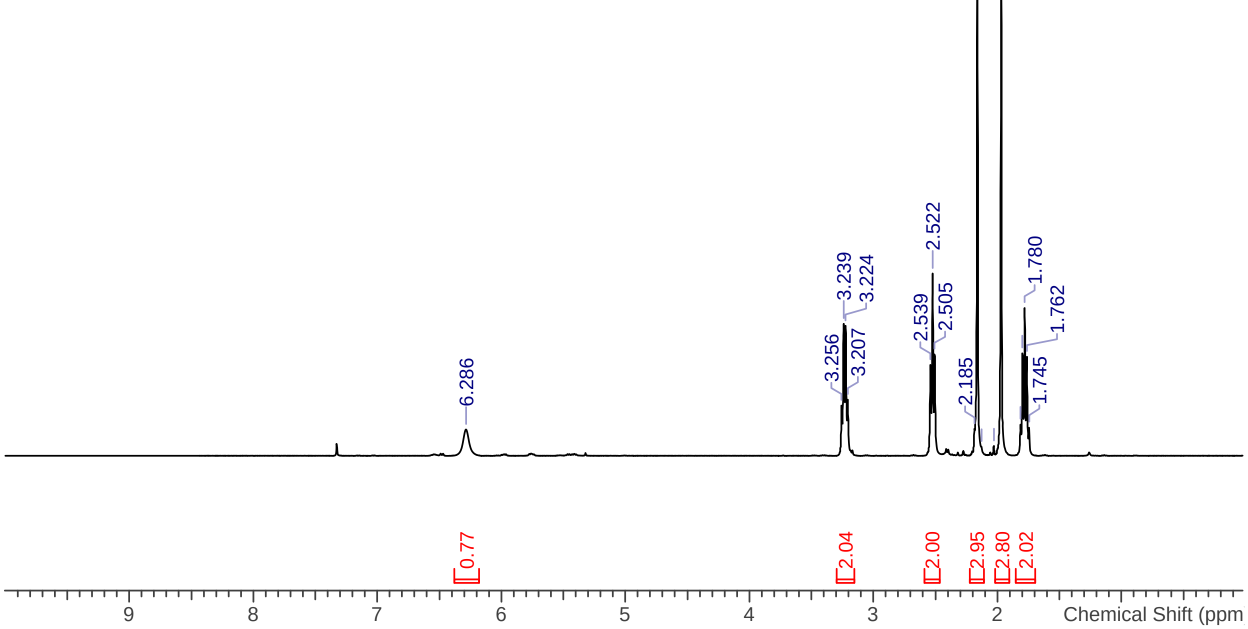

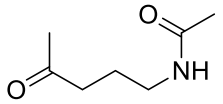

Fig. **S16**. ^{13}C NMR of **12**

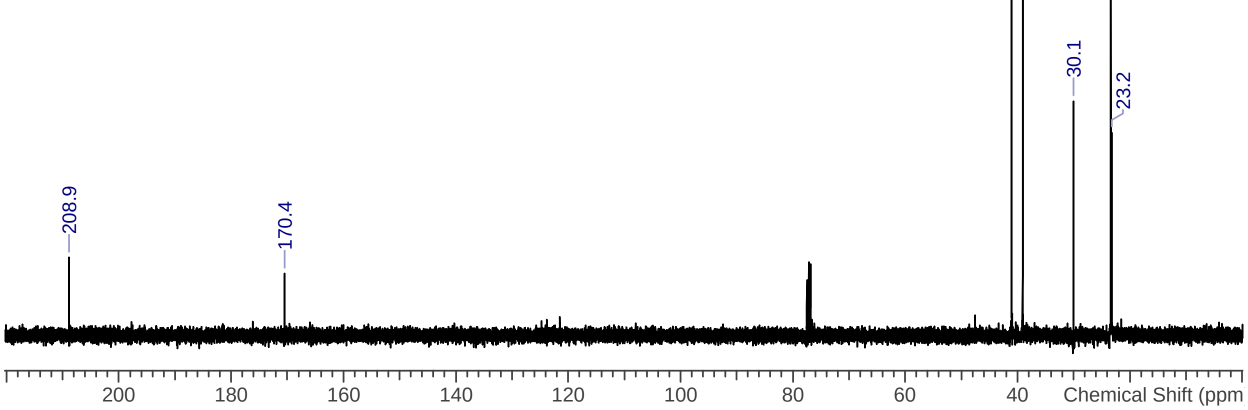

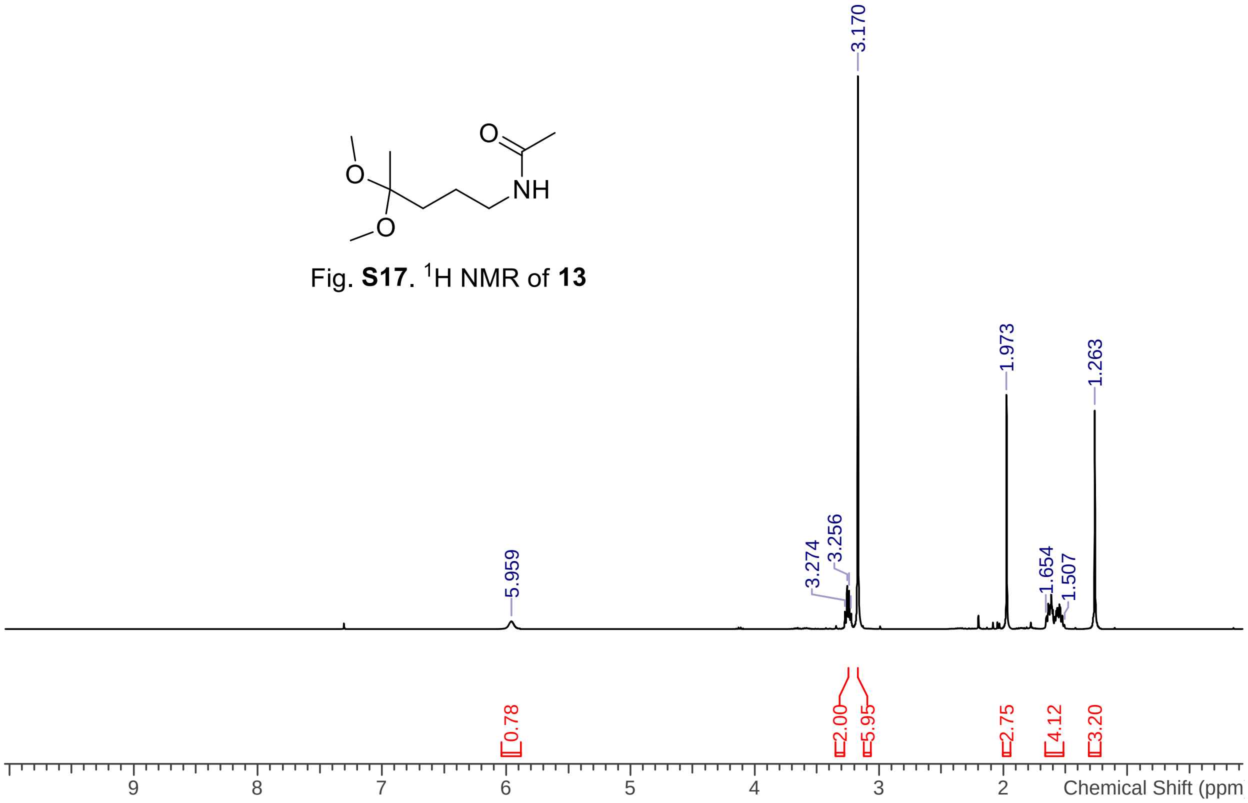

Fig. **S17**. ^{1}H NMR of **13**

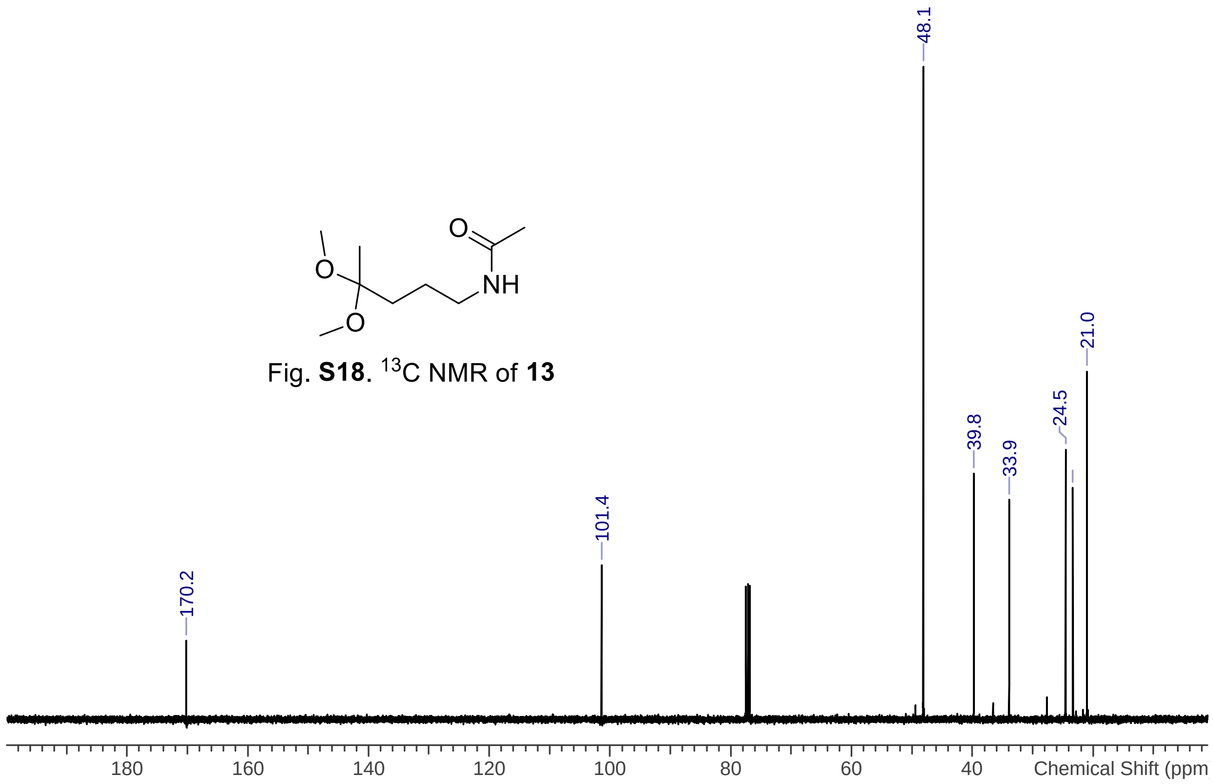

Fig. **S18**. ^{13}C NMR of **13**

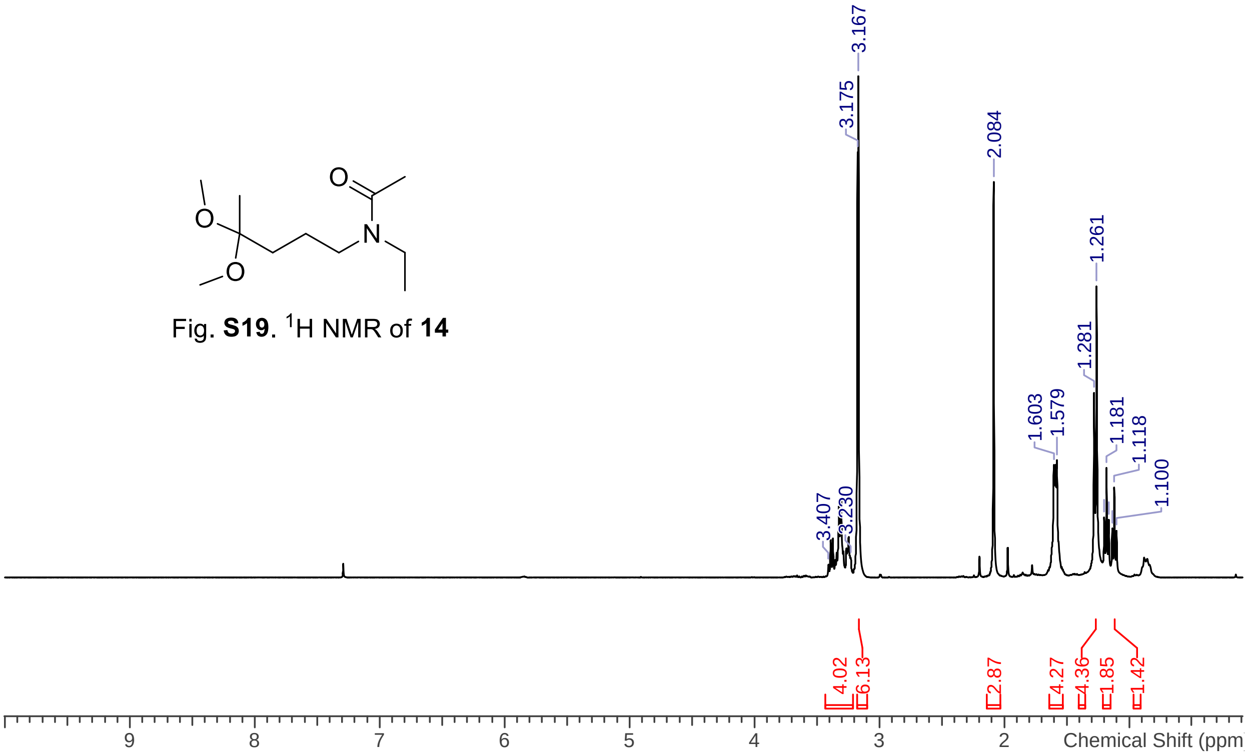

Fig. **S19**. ^{1}H NMR of **14**

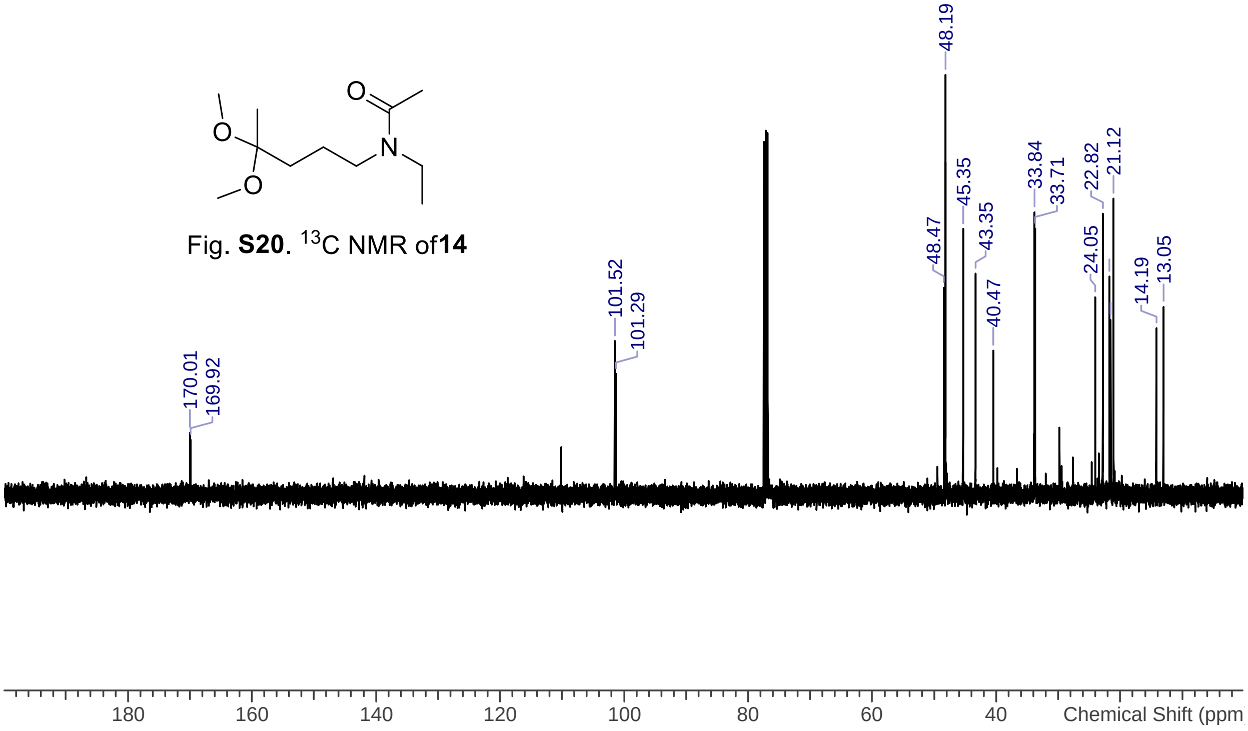

Fig. **S20**. ^{13}C NMR of**14**

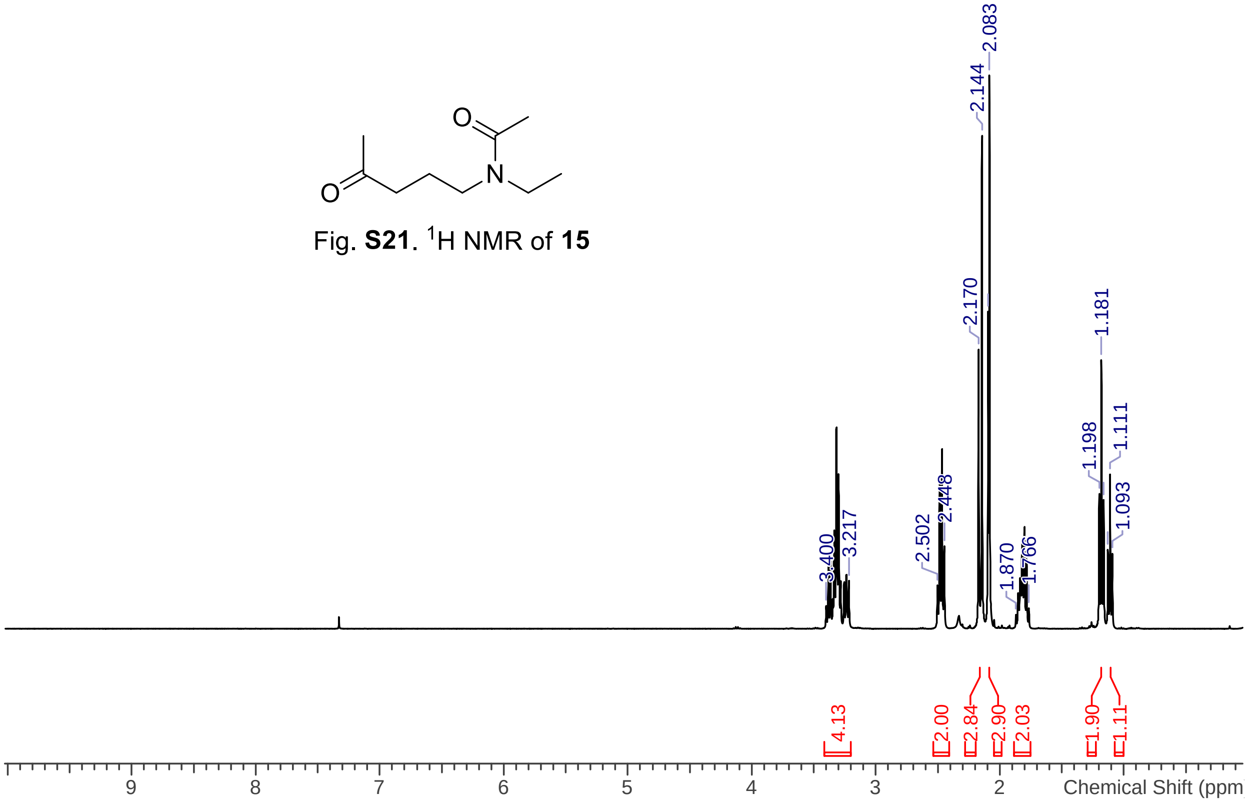

Fig. **S21**. ^{1}H NMR of **15**

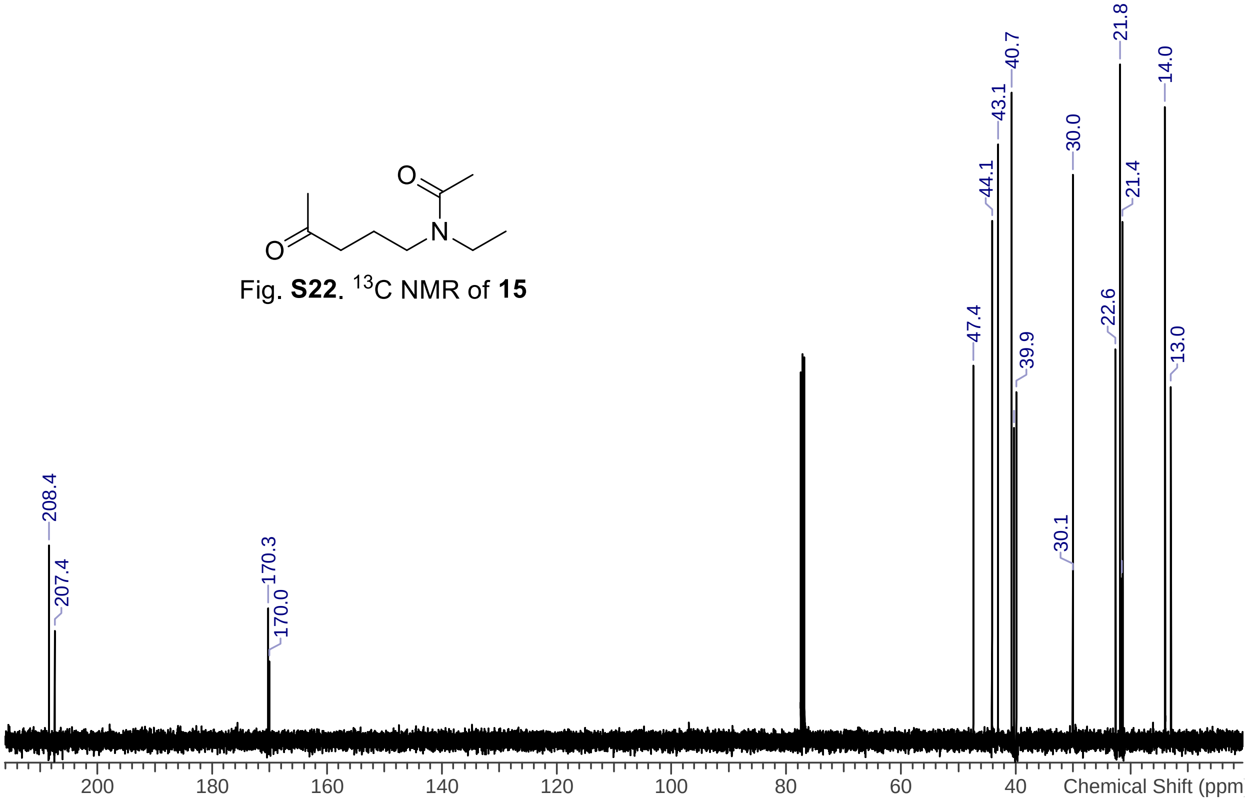

Fig. **S22**. ^{13}C NMR of **15**

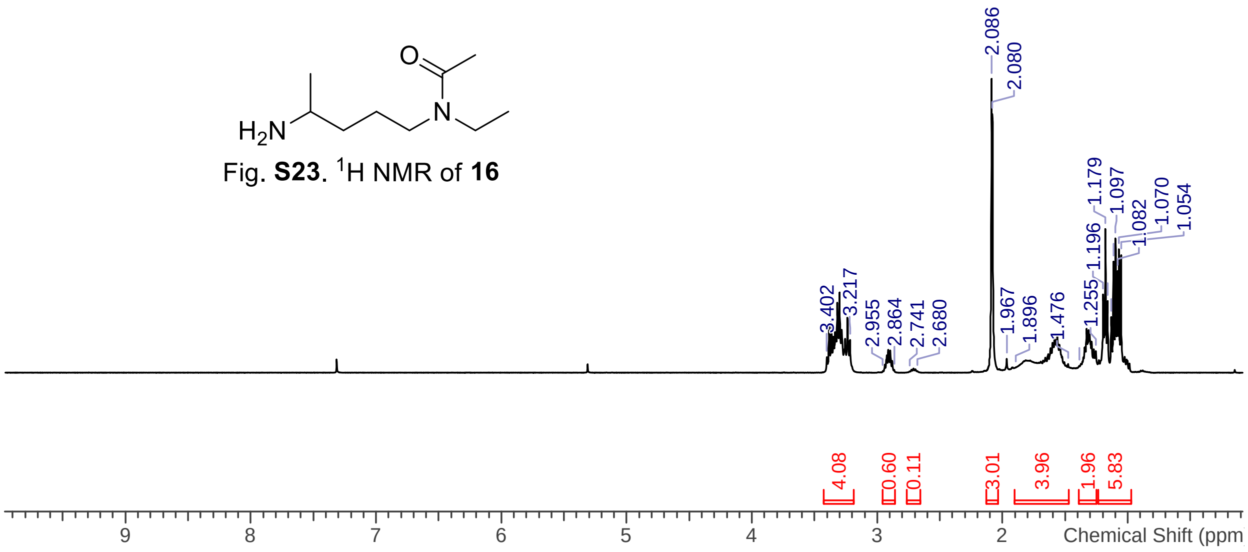

Fig. **S23**. ^{1}H NMR of **16**

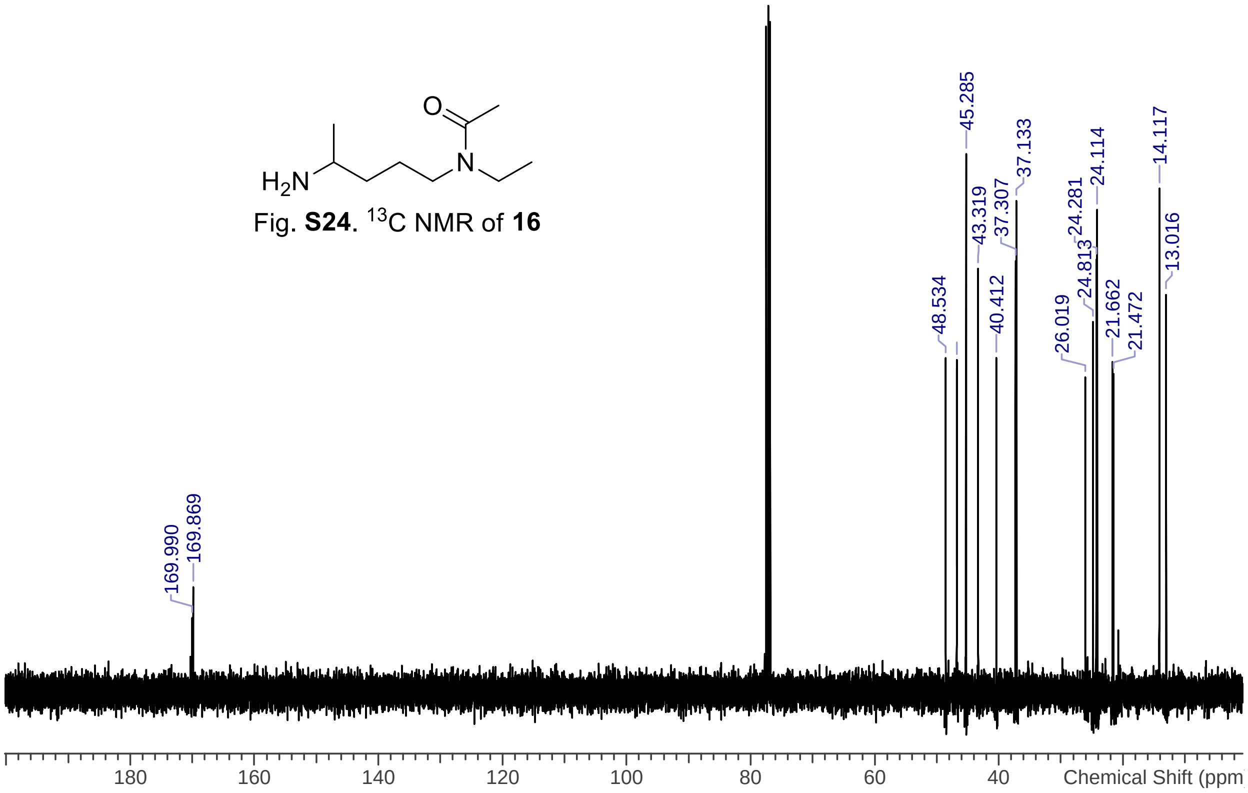

Fig. **S24**. ^{13}C NMR of **16**

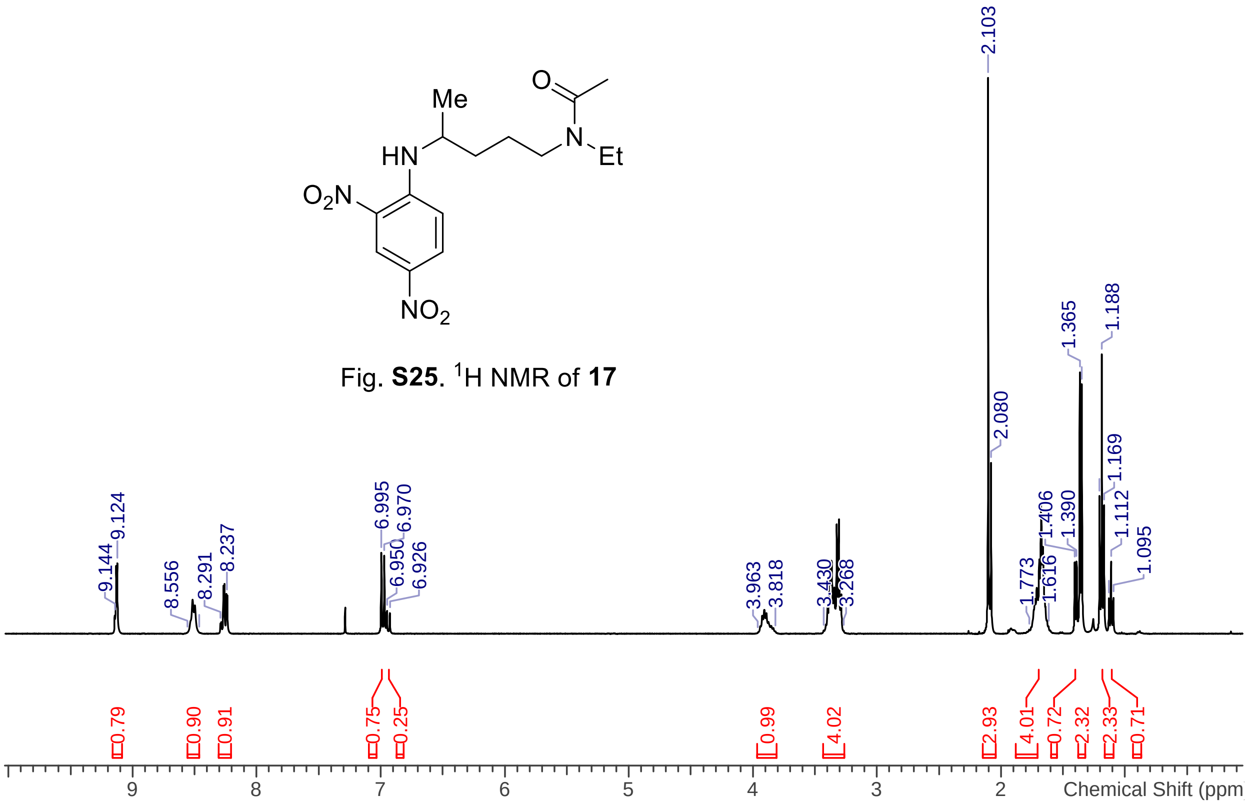

Fig. **S25**. ^{1}H NMR of **17**

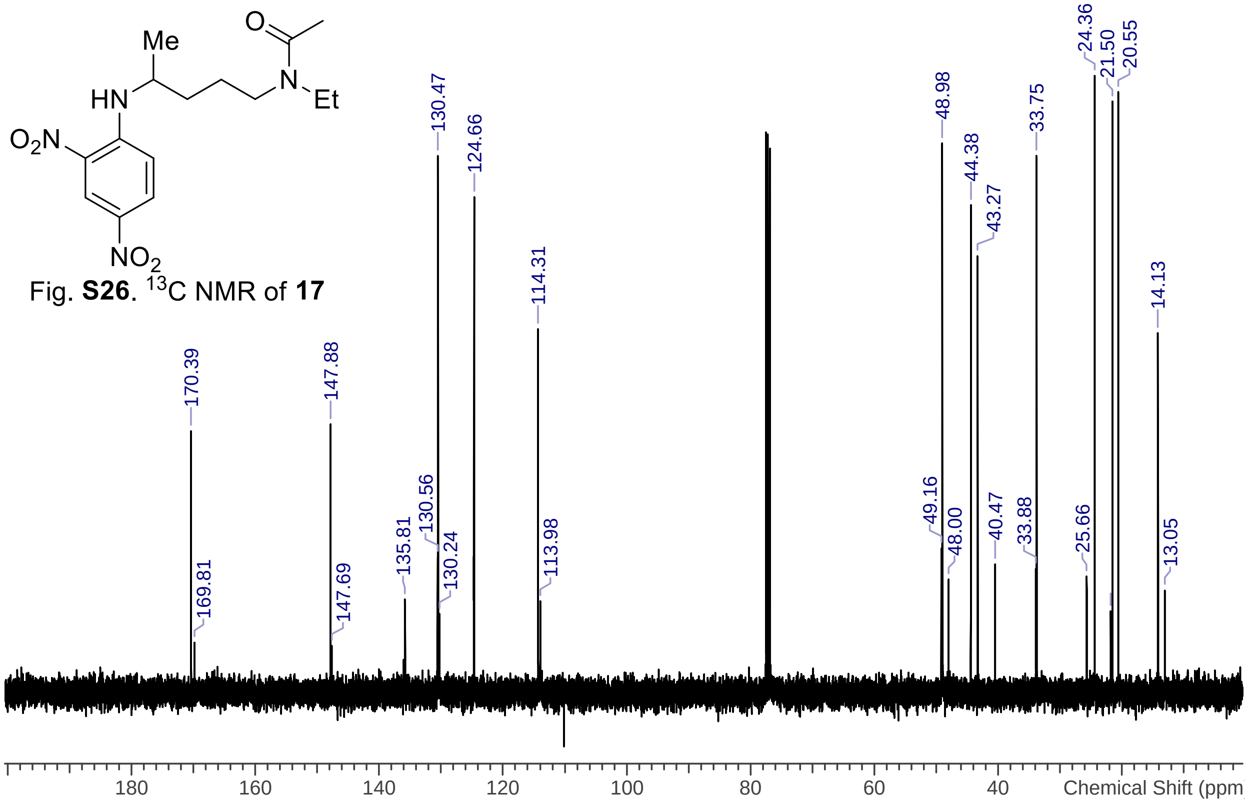

Fig. **S26**. ^{13}C NMR of **17**

Biochemical Data

Table S1 shows the data of biological tests presented in Table 1 of the manuscript. The numbers in the first column are the concentrations of tested drug candidates in μM. The numbers listed in other columns are the percent intensities relative to the blank samples, in which the concentrations of tested drugs are zero. "L-15" means L-15 media used for cell culture. "L-15 + G" means L-15 plus glucose media used for cell culture.

Table S1. Data of biological tests.

Compound	**1**		**2**		**8**	
(μM)	L-15	L-15 + G	L-15	L-15 + G	L-15	L-15 + G
0	100.0	100.0	100.0	100.0	100.0	100.0
6.25	94.2	111.7	104.8	108.0	102.4	107.1
12.5	72.5	114.1	110.9	116.6	89.2	106.3
25	26.0	100.7	107.2	112.9	91.2	102.5
50	18.0	77.3	91.8	108.7	57.2	100.4
100	20.5	47.5	61.7	81.8	21.7	83.4

Compound	**10**		**4**		**5**		**17**	
(μM)	L-15	L-15 + G	L-15	L-15 + G	L-15	L-15 + G	L-15	L-15 + G
0	100.0	100.0	100.0	100.0	100.0	100.0	100.0	100.0
12.5	109.4	113.3	100.5	104.8	100.1	112.6	96.2	107.0
25	106.5	122.8	97.5	106.0	111.2	120.7	52.4	109.6
50	91.2	117.4	92.4	104.1	79.6	112.4	14.0	99.0
100	101.1	114.2	80.2	99.9	72.8	114.0	12.0	53.1
200	88.6	109.3	48.2	77.5	59.3	100.1	11.2	24.7

References:

1. Yu, E.; Mangunuru, H. P. R.; Telang, N. S.; Kong, C. J.; Verghese, J.; Gilliland Iii, S. E.; Ahmad, S.; Dominey, R. N.; Gupton, B. F., High-yielding continuous-flow synthesis of antimalarial drug hydroxychloroquine. *Beilstein Journal of Organic Chemistry* **2018,** 14, 583-592.
